# La_2_O_3_ Filler's Stabilization of Residual Solvent in Polymer Electrolyte for Advanced Solid‐State Lithium‐Metal Batteries

**DOI:** 10.1002/smsc.202300017

**Published:** 2023-04-07

**Authors:** Yaping Zeng, Le Zhao, Jiaming Zhang, Qiuping Li, Dan Sun, Yu Ren, Yougen Tang, Guanhua Jin, Haiyan Wang

**Affiliations:** ^1^ Hunan Provincial Key Laboratory of Chemical Power Sources College of Chemistry and Chemical Engineering Central South University Changsha 410083 P. R. China; ^2^ Jiangsu Yeeli Technology Co., Ltd. Wuxi 214200 P. R. China; ^3^ College of Energy and Chemical Engineering Xinjiang Institute of Technology Aksu 843100 P. R. China

**Keywords:** composite electrolytes, ionic conductivity, La_2_O_3_ fillers, lithium-metal solid-state batteries, *N,N* -dimethylformamide

## Abstract

Polymer solid electrolytes (SEs) with high safety and flexibility are ideal for advanced lithium‐metal solid‐state batteries (SSBs). Among various polymer SEs, polyvinylidene fluoride*‐co*‐hexafluoropropylene (PVDF‐HFP) polymer SEs have gained increased attention for their high dielectric constants, high ionic conductivity, and excellent flexibility. However, severe side reactions at the interface caused by the decomposition of residual DMF solvent significantly reduce the cycle life of PVDF‐HFP‐based SSBs. Herein, La_2_O_3_ nanoparticles are used as new inorganic fillers to form a PVDF‐HFP/LiFSI/La_2_O_3_‐40% composite polymer electrolyte (PVDF‐HFP/La_2_O_3_ CPE). Benefiting from the interaction between La_2_O_3_ and *N,N*‐dimethylformamide (DMF) solvent molecules, the cell cycling stability is greatly improved. In addition, the PVDF‐HFP/LiFSI solid electrolyte (PVDF‐HFP SE) containing 40 wt% La_2_O_3_ has the highest ionic conductivity of 1.33 × 10^−3^ S cm^−1^ at 25 °C. It also exhibits a higher lithium‐ion transference number of 0.52 and lower polarization. The PVDF‐HFP/La_2_O_3_ CPE here ensures high ionic conductivity and stable interface chemistry in SSB, demonstrating a promising application potential.

## Graphical abstract

A novel La_2_O_3_ filler introduced into a polymer electrolyte to stabilize residual solvent ensures advanced solid‐state lithium‐metal batteries. The PVDF‐HFP/LiFSI solid electrolyte containing 40 wt% La_2_O_3_ has the highest ionic conductivity of 1.33 × 10^−3^ S cm^−1^ at 25 °C. Moreover, the full cell assembled with PVDF‐HFP/LiFSI/La_2_O_3_ presents greatly improved cycling stability, demonstrating a promising application potential.

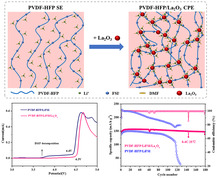

## 1 Introduction

Lithium‐ion batteries (LIBs) have been used in various fields and have significantly changed people's lives in the past few decades.^[^ ^1^ ^]^ However, the unprecedented demand for energy storage presents the development goals of high safety and high energy density. Compared to conventional LIBs, lithium‐metal SSBs are considered to be the most promising candidates for advanced high‐energy storage systems due to their nonleakage, wide electrochemical window, and many other merits.^[^ ^2^ ^]^ SEs are critical to developing advanced SSBs.

In recent years, various SEs including inorganic SEs, polymer SEs, and composites SEs have been intensively studied.^[^ ^3^ ^]^ Among them, inorganic SEs mainly include oxides and sulfides.^[^ ^4^ ^]^ Typical oxide SEs such as garnet‐type,^[^ ^5^ ^]^NASICON‐type,^[^ ^6^ ^]^ perovskite‐type,^[^ ^7^ ^]^ and anti‐perovskite‐type^[^ ^8^ ^]^ electrolytes have high ionic conductivity (>10^−4^ S cm^−1^) and relatively stable structure.^[^ ^3^, ^5^, ^9^ ^]^ However, oxide SEs have high synthesis temperatures, high fabrication cost, high rigidity, and poor interfacial contact with the cathode and anode.^[^ ^4^, ^5^ ^]^. Currently, the more studied sulfide SEs are Li_10_GeP_2_S_12_,^[^ ^10^ ^]^ Li_2_S‐P_2_S_5_,^[^ ^11^ ^]^ and Li_6_PS_5_X (X = Cl, Br, I).^[^ ^12^ ^]^Compared with oxide SEs, sulfide SEs have higher ionic conductivity, lower synthesis temperature, and better flexibility.^[^ ^13^ ^]^ However, sulfide SEs are susceptible to reduction at lower voltages and have poor electrochemical stability.^[^ ^14^ ^]^ Tremendous efforts such as elemental doping,^[^ ^15^ ^]^ optimization of preparation methods,^[^ ^16^ ^]^interfacial modification,^[^ ^17^ ^]^ and exploitation of novel electrolyte systems have been made to optimize inorganic SE systems, and the development of inorganic SEs has made breakthrough progress. However, the interface issues between inorganic SEs with electrodes still severely limit their practical applications.^[^ ^18^ ^]^ Fortunately, polymer‐based SEs with good electrode contact stability and excellent processing properties offer great promise for practical applications of SSBs.^[^ ^19^ ^]^ The main polymer SEs that have been reported include polyethylene oxide (PEO),^[^ ^20^ ^]^ polyacrylonitrile (PAN),^[^ ^21^ ^]^ poly(vinylidene fluoride) (PVDF),^[^ ^22^ ^]^ poly(methyl methacrylate) (PMMA),^[^ ^23^ ^]^ and poly(propylene carbonate) (PPC).^[^ ^24^ ^]^ However, the inherent issues such as low room‐temperature ionic conductivity, poor mechanical properties, and poor interfacial stability of polymeric SEs have greatly limited their further development.^[^ ^25^ ^]^ Therefore, the modification of polymer SEs is mainly focused on improving the ionic conductivity,^[^ ^26^ ^]^ enhancing the mechanical properties,^[^ ^27^ ^]^ and improving the chemical stability at the cathode/anode interface.^[^ ^28^ ^]^ The specific modification strategies include polymer blending,^[^ ^29^ ^]^ grafting,^[^ ^30^ ^]^ crosslinking,^[^ ^31^ ^]^ and adding inorganic fillers. Among them, it is an effective strategy to add inorganic fillers to prepare composite SEs. The fillers are mainly divided into conductive active fillers (Li_7_La_3_Zr_2_O_12_‐based SE,^[^ ^32^ ^]^ Li_0.29_La_0.57_TiO_3_,^[^ ^33^ ^]^Li_1.5_Al_0.5_Ti_1.5_(PO_4_)_3_,^[^ ^34^ ^]^ Li_10_GeP_2_S_12_,^[^ ^35^ ^]^ etc.) and nonconductive inert fillers (ZrO_2_,^[^ ^36^ ^]^ Al_2_O_3_,^[^ ^37^ ^]^ TiO_2_,^[^ ^38^ ^]^ etc.). Adding fillers can enhance the ionic conductivity of polymeric SEs. The addition of filler reduces the orderliness of the polymer to form more amorphous regions, thus enhancing the ionic conductivity.^[^ ^39^ ^]^ In addition, the existence of channels for ion transport on the surface of the filler particles accelerates ion transport.^[^ ^40^ ^]^ Simultaneously, adding fillers can also significantly enhance the mechanical strength, broaden the electrochemical voltage window of polymeric SEs, and improve the interfacial stability between electrolytes and electrodes.

In polymer SE systems, PVDF‐HFP polymer electrolyte is attracting great attention for their high dielectric constants, good electrochemical stability, and thermal stability.^[^ ^41^ ^]^ Note that during the preparation of PVDF‐HFP polymer electrolyte even after sufficient thermal treatment, there is still very tiny residual DMF solvent within the polymer matrix, which is not dissociative but will contribute good ionic conductivity to the electrolyte.^[^ ^42^ ^]^ Nevertheless, owing to the interfacial side reactions and other interfacial issues caused by the decomposition of DMF at high voltages, the residual DMF solvent will drastically reduce the cycling stability of the cell.^[^ ^43^ ^]^ To address this issue, great efforts have focused on understanding the role of DMF in polymers, regulating DMF residues, and developing novel solvents.[^43^, ^44^] In addition, adding inorganic SEs, for example, Li_1.4_Al_0.4_Ti_1.6_(PO_4_)_3_ (LATP)^[^ ^45^ ^]^ and Li_6.75_La_3_Zr_1.75_Ta_0.25_O_12_ (LLZTO),^[^ ^46^ ^]^ seems a good strategy to improve interfacial stability since interfacial side reactions of fillers with residual DMF solvents can be suppressed. However, these aforementioned inorganic electrolytes are complicated to synthesize and very expensive. Therefore, it is of great interest to explore inexpensive inorganic fillers for achieving stable PVDF‐HFP polymer. Zhang et al.^[^ ^46^ ^]^ proposed that La atoms in LLZTO would form complexes with N atoms and C═O group of DMF solvent molecules. Thus La‐based compounds may have potential for stabilizing DMF solvents. However, there are no relevant studies on the effect of La‐based oxides on PVDF‐HFP‐based SEs.

In this work, we discover that La_2_O_3_ nanoparticles can be incorporated into the polymer electrolyte as a useful inorganic filler, and a stable CPE composed of PVDF‐HFP matrix with La_2_O_3_ nanoparticles and LiFSI is developed. Surprisingly, La_2_O_3_ nanoparticles have a good adsorption effect on DMF molecules, which can effectively inhibit its decomposition at high voltages, thus resulting in enhanced electrochemical stability of the CPE. Moreover, with the addition of La_2_O_3_ nanoparticles, the obtained PVDF‐HFP/La_2_O_3_ CPE exhibits better mechanical properties and thermal stability. More importantly, the SSB assembled based on this CPE achieves excellent battery performance.

## 2 Results and Discussion

**Figure 1:**
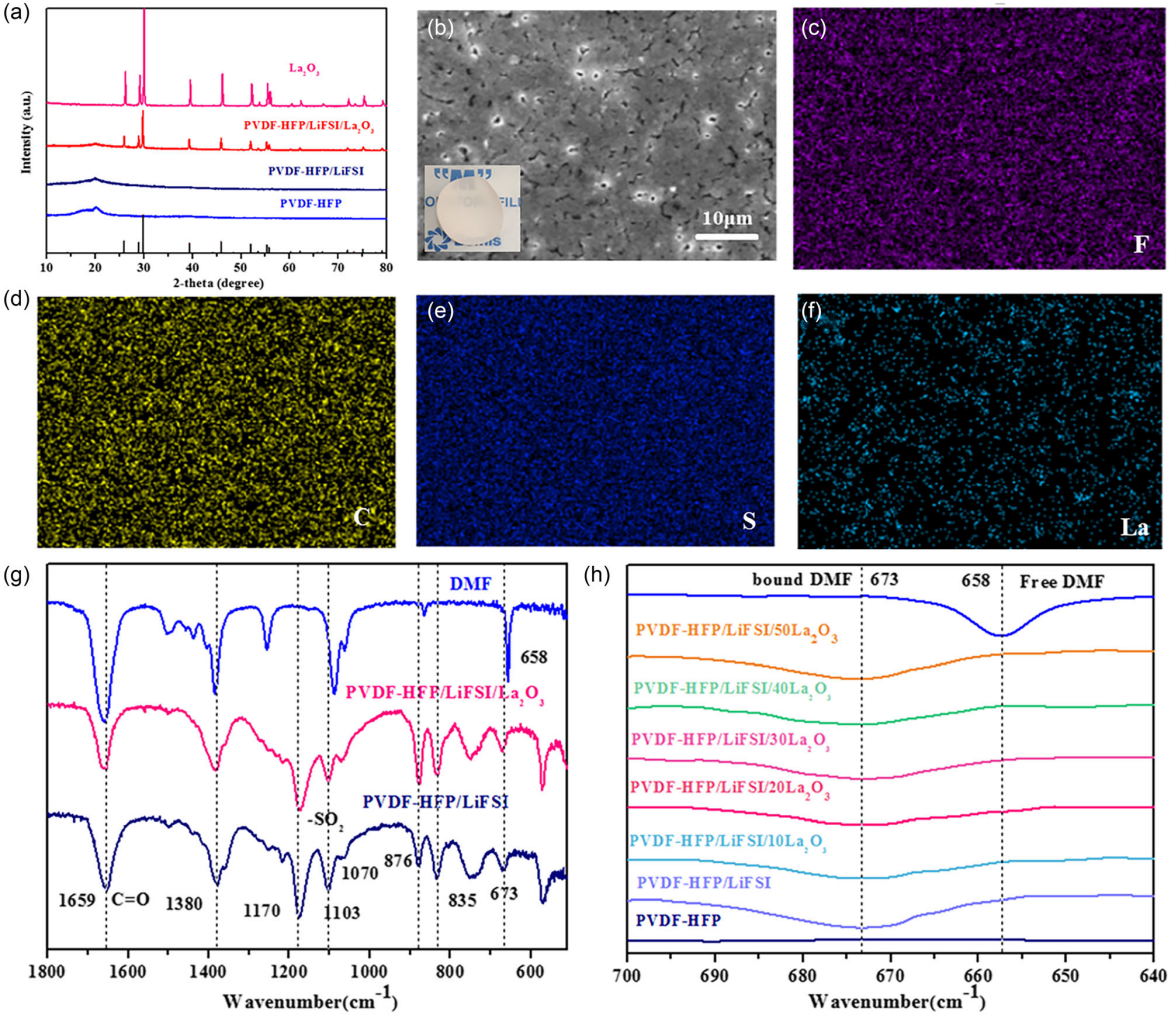
a) XRD patterns of La_2_O_3_, pure PVDF‐HFP, PVDF‐HFP SE, and PVDF‐HFP/La_2_O_3_ CPE. b–f) Surface SEM image of PVDF‐HFP/La_2_O_3_ CPE and the corresponding EDS mappings. g–h) FTIR spectra of DMF molecule, pure PVDF‐HFP, PVDF‐HFP SE, and PVDF‐HFP/La_2_O_3_ CPEs.

**Figure** **1**a displays the X‐ray diffraction (XRD) patterns of prepared electrolyte membranes. The diffraction peaks include all the characteristic peaks of the PVDF‐HFP matrix and La_2_O_3_ nanoparticles without other impurity peaks, indicating that the inorganic filler and polymer matrix was successfully compounded and no new phase was formed. In addition, the comparison in the XRD shows that the intensity of the diffraction peaks of La_2_O_3_ decreases after being embedded in the PVDF‐HFP SE, which means a reduction in the crystallinity degree of La_2_O_3_. More importantly, the crystallinity of PVDF‐HFP SE decreases with the addition of La_2_O_3_, which indicates that adding La_2_O_3_ filler and LiFSI can increase the amorphous area of the PVDF‐HFP matrix. The scanning electron microscopy (SEM) image of PVDF‐HFP/La_2_O_3_ CPE is presented in Figure 1b. As shown, the membrane surface is relatively dense and smooth, suggesting that La_2_O_3_ particles are uniformly distributed within the PVDF‐HFP matrix, which can facilitate internal Li^+^ transport. As further evidenced by the energy‐dispersive spectral (EDS Figure 1c–f **)** mapping images of the PVDF‐HFP/La_2_O_3_ CPE, it is obvious that the corresponding elements are distributed evenly. The thickness of this CPE is about 100 mm presented from its cross‐sectional image in Figure S1, Supporting Information. The Fourier‐transform infrared (FTIR) spectra of obtained membranes are given in Figure g–h. The characteristic peaks at 1659 and 1380 cm^−1^ represent C═O vibration and –CH_3_ in DMF molecule.^[^ ^45^ ^]^ The peaks observed at 1170 and 1070 cm^−1^ are ascribed to –CF_2_ absorption peak in PVDF‐HFP matrix, and the peaks at 835 and 876 cm^−1^ are assigned to the amorphous phase of PVDF‐HFP.^[^ ^47^, ^48^ ^]^ The bands at 658 and 673 cm^−1^ belong to uncoordinated O═C–N (free DMF) and coordinated [Li(DMF)_*x*_]^+^ (bound DMF), respectively.^[^ ^46^ ^]^ It should be noted that the band at 673 cm^−1^ in obtained membranes demonstrates that the residual DMF solvent exists in the electrolyte as bound DMF.

**Figure 2:**
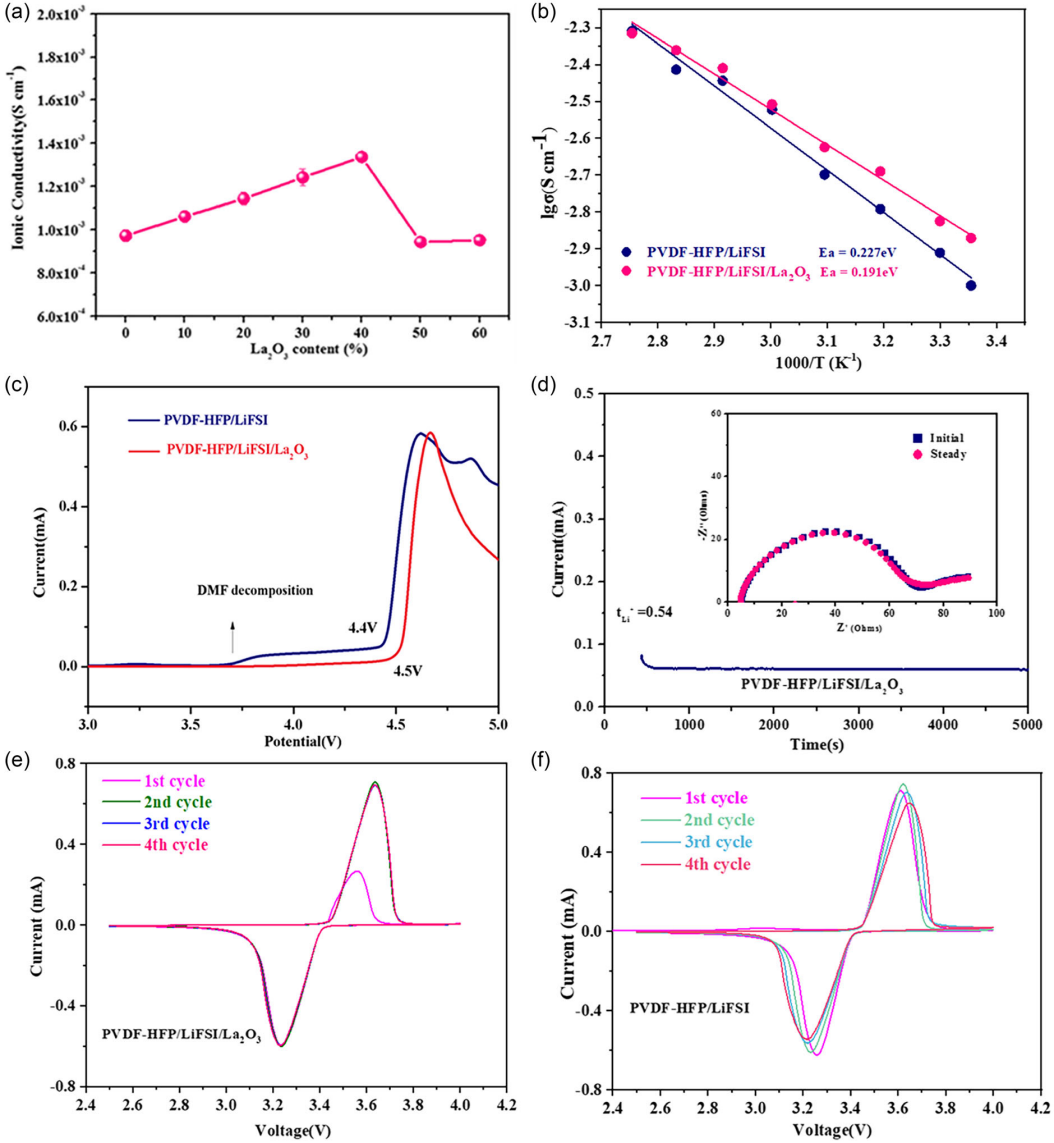
a) Ionic conductivity of obtained PVDF‐HFP/La_2_O_3_ CPEs with different La_2_O_3_ contents. Values are expressed as mean ± SD (*n* = 3). b) Arrhenius plots of ionic conductivities of the PVDF‐HFP SE and PVDF‐HFP/ La_2_O_3_ CPE. c) LSV plots of PVDF‐HFP SE and PVDF‐HFP/La_2_O_3_ CPE. d) Direct current polarization result for the Li||PVDF‐HFP/La_2_O_3_ CPE||Li symmetrical cell and its AC impedance curves before and after polarization (inset). e,f) CV curves of the LFP||PVDF‐HFP/La_2_O_3_ CPE||Li cell (e) and the LFP||PVDF‐HFP SE||Li cell (f).

**Figure 3:**
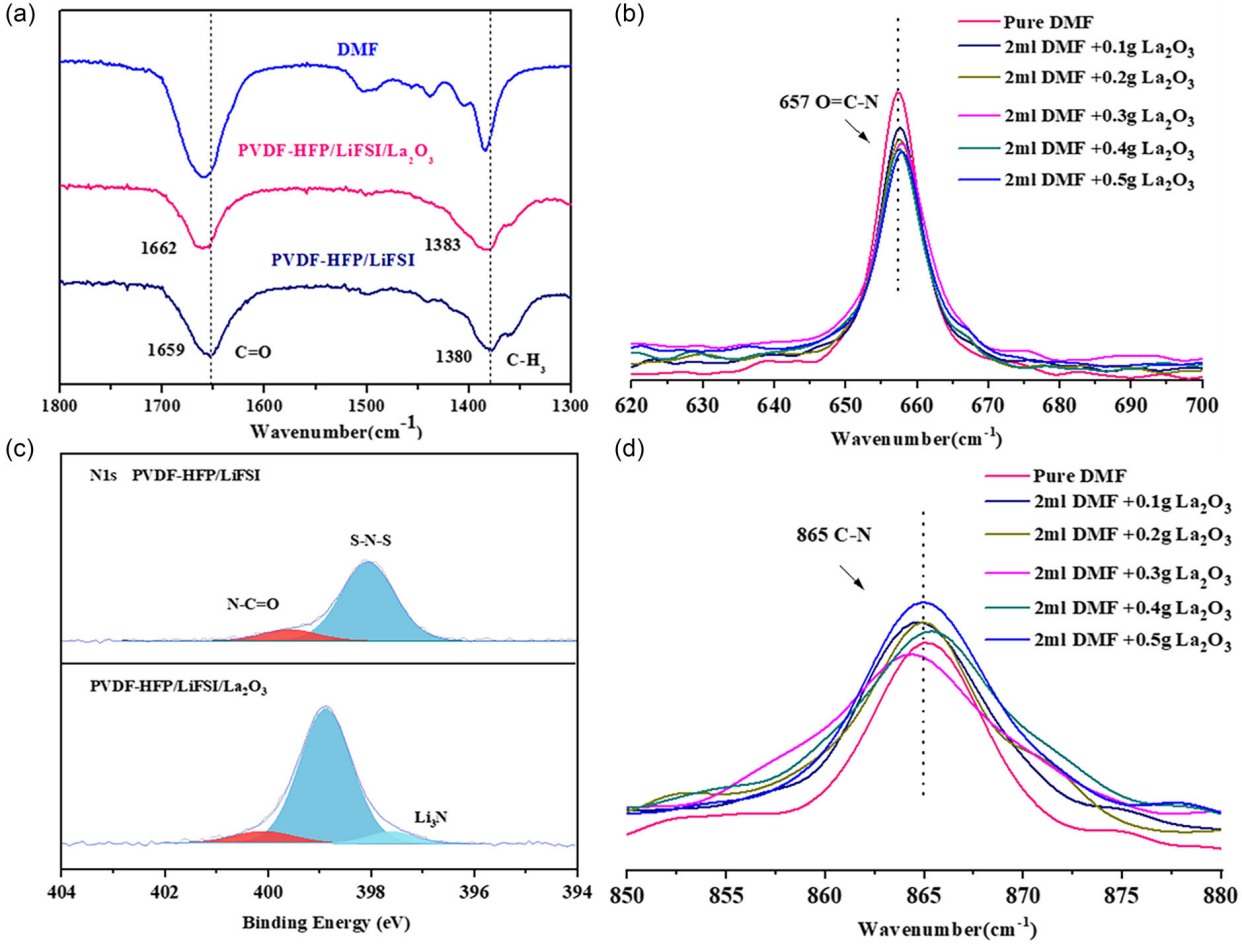
a) FTIR spectra of DMF, PVDF‐HFP SE, and PVDF‐HFP/La_2_O_3_ CPE. b,d) FTIR spectra of DMF and DMF/La_2_O_3_ mixtures at the wavenumbers of 620–700 cm^−1^ (b) and 850–880 cm^−1^ (d). c) XPS spectra for N1s PVDF‐HFP SE, and PVDF‐HFP/La_2_O_3_ CPE.

Ionic conductivity is a vital property of SEs. To investigate the effect of La_2_O_3_ nanoparticles addition on the ionic conductivity of PVDF‐HFP SE, the ionic conductivity of obtained CPEs with different La_2_O_3_ contents (0%–60%) was measured, and the results are shown in **Figure** **2**a. With increasing the proportion of La_2_O_3_, the CPE containing 40% La_2_O_3_ exhibits the highest room‐temperature ionic conductivity of 1.33 × 10^−3^ S cm^−1^, which is higher than that of PVDF‐HFP SE without La_2_O_3_ nanoparticles (9.74 × 10^−4^ S cm^−1^). This could benefit from the reduced crystallinity of the PVDF‐HFP matrix with the addition of La_2_O_3_, which can be further confirmed by differential scanning calorimetry (DSC).^[^ ^49^ ^]^ However, excessive La_2_O_3_ filler will hinder Li^+^ conduction and present a decrease in the ionic conductivity of PVDF‐HFP/La_2_O_3_ CPEs, which is attributed to the aggregation of nanoparticles. The DSC curves of PVDF‐HFP SE and PVDF‐HFP/La_2_O_3_ CPE were examined **(**Figure S2, Supporting Information). The endothermic peak of PVDF‐HFP SE appears at 169 °C, which represents the melting temperature (*T*_m_) of PVDF‐HFP. When adding La_2_O_3_, the peaks decrease significantly to 157 °C, which reveals the reduced crystallization stability of PVDF‐HFP.^[^ ^47^ ^]^ The impedance spectra of PVDF‐HFP SE and PVDF‐HFP/La_2_O_3_ CPE at different temperatures are presented in Figure S3, Supporting Information. The Arrhenius plots of PVDF‐HFP SE and PVDF‐HFP/La_2_O_3_ CPE are shown in Figure 2b, from which the corresponding activation energy is calculated to be 0.227 and 0.191 eV, respectively. The PVDF‐HFP/La_2_O_3_ CPE shows relatively lower activation energy, indicating the facilitation of Li^+^ migration. **Figure** **3**d and S4, Supporting Information, show the direct current polarization and the AC impedance curves before and after polarization of the PVDF‐HFP/La_2_O_3_ CPE and PVDF‐HFP SE. The calculated lithium transference number ($t_{{\text{Li}}^{+}}$) of PVDF‐HFP/La_2_O_3_ CPE reaches 0.54, much higher than that of PVDF‐HFP SE (0.36). The higher $t_{{\text{Li}}^{+}}$ of the PVDF‐HFP/La_2_O_3_ CPE indicates that the addition of La_2_O_3_ filler can effectively immobilize the movement of FSI^−^ and promote the effective migration of Li^+^.^[^ ^50^ ^]^ The linear scanning voltammetry (LSV) curve shows that with the introduction of La_2_O_3_ nanoparticles, the decomposition of DMF at 3.8 V in PVDF‐HFP SE was greatly suppressed and the electrochemical stability window was extended from 4.4 to 4.5 V (Figure 2c), indicating its better compatibility with high‐voltage lithium batteries.^[^ ^48^ ^]^ The cyclic voltammetry (CV) measurements of the assembled LiFePO_4_ (LFP)||PVDF‐HFP/La_2_O_3_ CPE||Li (Figure 2e) and LFP||PVDF‐HFP SE||Li (Figure 2f) were performed at room temperature from 2.5 to 4.0 V. The CV curves of LFP||PVDF‐HFP/La_2_O_3_ CPE||Li are basically overlapped, indicating the superior electrochemical stability of the composite electrolyte membrane.^[^ ^51^ ^]^

Good mechanical strength is an important property of SEs. The tensile strengths of obtained PVDF‐HFP SE and PVDF‐HFP/La_2_O_3_ CPE were investigated and are presented in Figure S5, Supporting Information. Compared with PVDF‐HFP SE, the tensile strength of PVDF‐HFP/La_2_O_3_ CPE is remarkably increased. Good thermal stability is critical for SEs. Thermogravimetric analysis (TGA) was performed to evaluate the thermal stability of pure PVDF‐HFP, PVDF‐HFP SE, and PVDF‐HFP/La_2_O_3_ CPE, respectively. As presented in TGA results (Figure S6, Supporting Information), the slight weight loss of the membranes before 140 °C is ascribed to the captured moisture, and the evaporation of residual DMF solvent occurs at 140–200 °C. Therefore, the DMF solvent residues of electrolyte membranes are less than 10%. The apparent weight loss at 200–310 °C is derived from the thermal degradation of LiFSI. The complete thermal decomposition of PVDF‐HFP/La_2_O_3_ CPE occurs at 310 °C.^[^ ^47^ ^]^ Nevertheless, the thermal stability of PVDF‐HFP/La_2_O_3_ CPE is still considerable for applications in lithium batteries.

To elucidate the interaction between La_2_O_3_ and DMF molecules, FTIR and X‐ray photoelectron spectroscopy (XPS) of PVDF‐HFP SE and PVDF‐HFP/La_2_O_3_ CPE were performed (Figure 3). The peaks at 1659 and 1380 cm^−1^ are assigned to C═O and –CH_3_ of DMF solvent (Figure 3a).^[^ ^45^ ^]^ Clearly, the C═O and –CH_3_ peaks of DMF are weakened and shifted to 1662 and 1383 cm^−1^ with the addition of La_2_O_3_, respectively, which is attributed to the strong interactions of La_2_O_3_ with DMF.^[^ ^45^ ^]^ Furthermore, FTIR spectra of La_2_O_3_ and pure DMF solvent mixtures were employed to demonstrate the interaction between La_2_O_3_ and DMF molecules (Figure 3b,d). With the increase of La_2_O_3_, the peak intensity of free DMF decreases and the peak position is significantly shifted, which means that La_2_O_3_ interacts with the DMF molecule.^[^ ^46^ ^]^ The XPS measurements of PVDF‐HFP SE and PVDF‐HFP/La_2_O_3_ CPE further demonstrate the interaction between La_2_O_3_ and DMF molecule. The N1s spectrum peaks appear at 398.86 and 400.69 eV, corresponding to S—N—S bonds in LiFSI^[^ ^52^ ^]^ and N—C═O in DMF,^[^ ^42^ ^]^ respectively (Figure 3c). With the La_2_O_3_ addition, N1s peaks correspond to S—N—S bonds, and N—C═O shift to 399.82 and 401.27 eV, respectively. The results show that the interaction between La_2_O_3_ and DMF occurs through N—C═O.

To further verify the adsorption effect of La_2_O_3_ on DMF molecules, the adsorption energy was calculated by density functional theory (DFT) calculations (Figure S7, Supporting Information). The adsorption energy of DMF on La_2_O_3_ is −0.637 eV, much higher than that on PVDF‐HFP (−0.129 eV), revealing that La_2_O_3_ has strong adsorption with residual DMF solvent in CPE.

**Figure 4:**
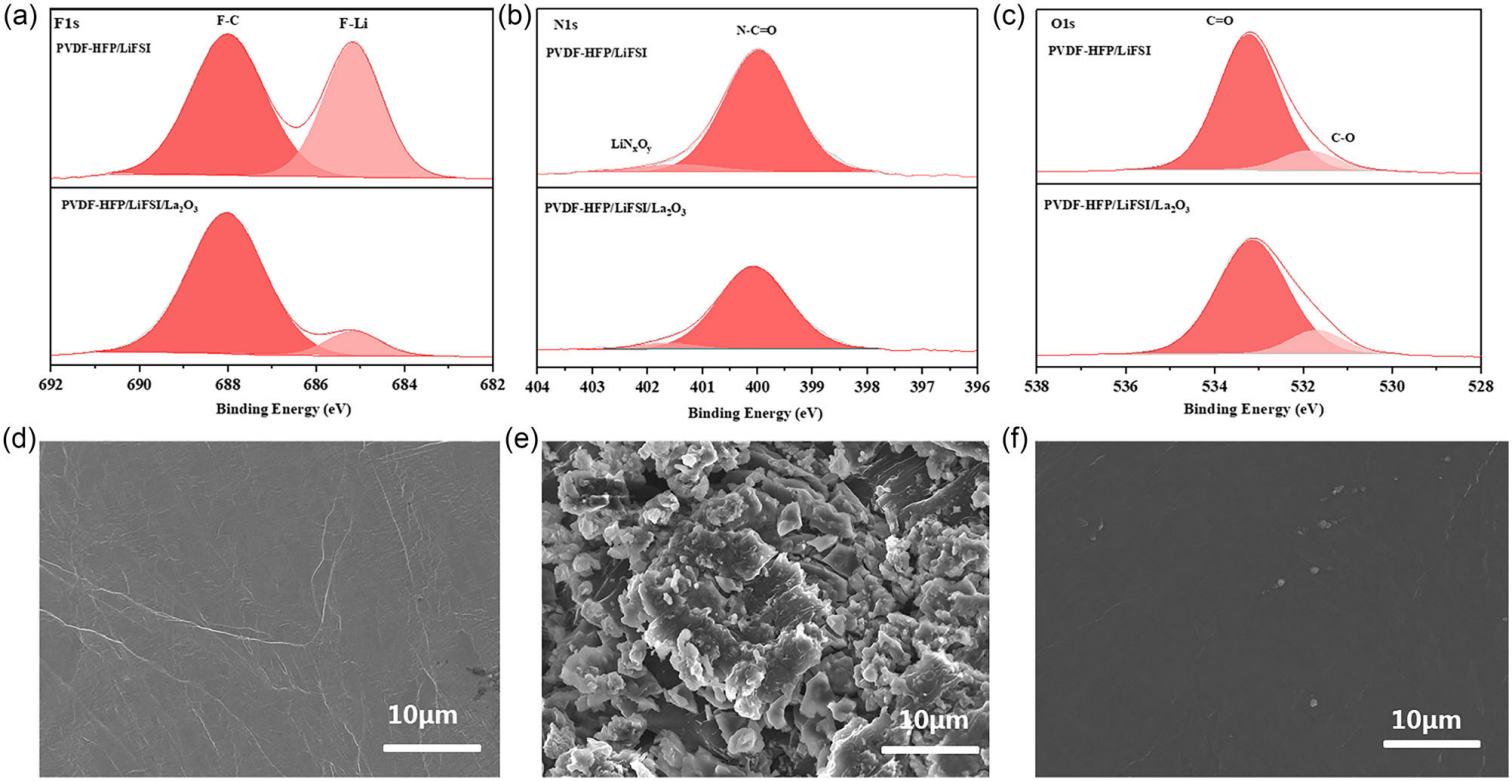
a–c) XPS spectra for F1s (a), N1s (b), and O1s (c) of cycled LFP cathode and d–f) SEM images of fresh (d) and cycled (e,f) lithium metal anodes with PVDF‐HFP (e) SE and PVDF‐HFP/La_2_O_3_ CPE (f).

In addition, the XPS spectra of LFP cathodes with PVDF‐HFP SE and PVDF‐HFP/La_2_O_3_ CPE after 30 cycles were also characterized to describe the chemical environment at the LFP cathode interface. The Li–F (685.1 eV) appears in the F 1s (**Figure** **4**a) derived from the decomposition of LiFSI.^[^ ^53^ ^]^ The spectrum peak at 688.0 eV is assigned to C–F attributed to the decomposition of PVDF‐HFP.^[^ ^54^ ^]^ The C═O (533 eV) and C—O (531 eV) of Li_2_CO_3_ presented in O1s are attributed to the decomposition of PVDF‐HFP and DMF (Figure 4c).^[^ ^55^ ^]^ The N—C=O (400.01 eV)^[^ ^42^ ^]^ and Li_*x*_NO_*y*_ (401.5 eV)^[^ ^48^ ^]^ detected in N1s spectra are ascribed to DMF solvent and the hysteretic decomposition of DMF, respectively (Figure 4b). Compared with PVDF‐HFP SE, the significantly decreased peak intensities of F1s, O1s, and N1s spectra using PVDF‐HFP/La_2_O_3_ CPE indicate that the side reactions of the cathode interface are greatly inhibited.^[^ ^45^ ^]^ Figure 4d,e shows the SEM images of fresh lithium metal, and the cycled lithium‐metal anode after 30 cycles of LFP||PVDF‐HFP SE||Li, and LFP||PVDF‐HFP/La_2_O_3_ CPE||Li, respectively. The lithium‐metal anode using PVDF‐HFP SE exhibits extremely rough surfaces and irregular breakage, indicating severe lithium‐related side reactions in the battery. Compared with LFP||PVDF‐HFP SE||Li, the lithium metal in PVDF‐HFP/La_2_O_3_ CPE demonstrates much smoother morphology, which indicates much better interfacial stability of LFP||PVDF‐HFP/La_2_O_3_ CPE||Li battery.

**Figure 5:**
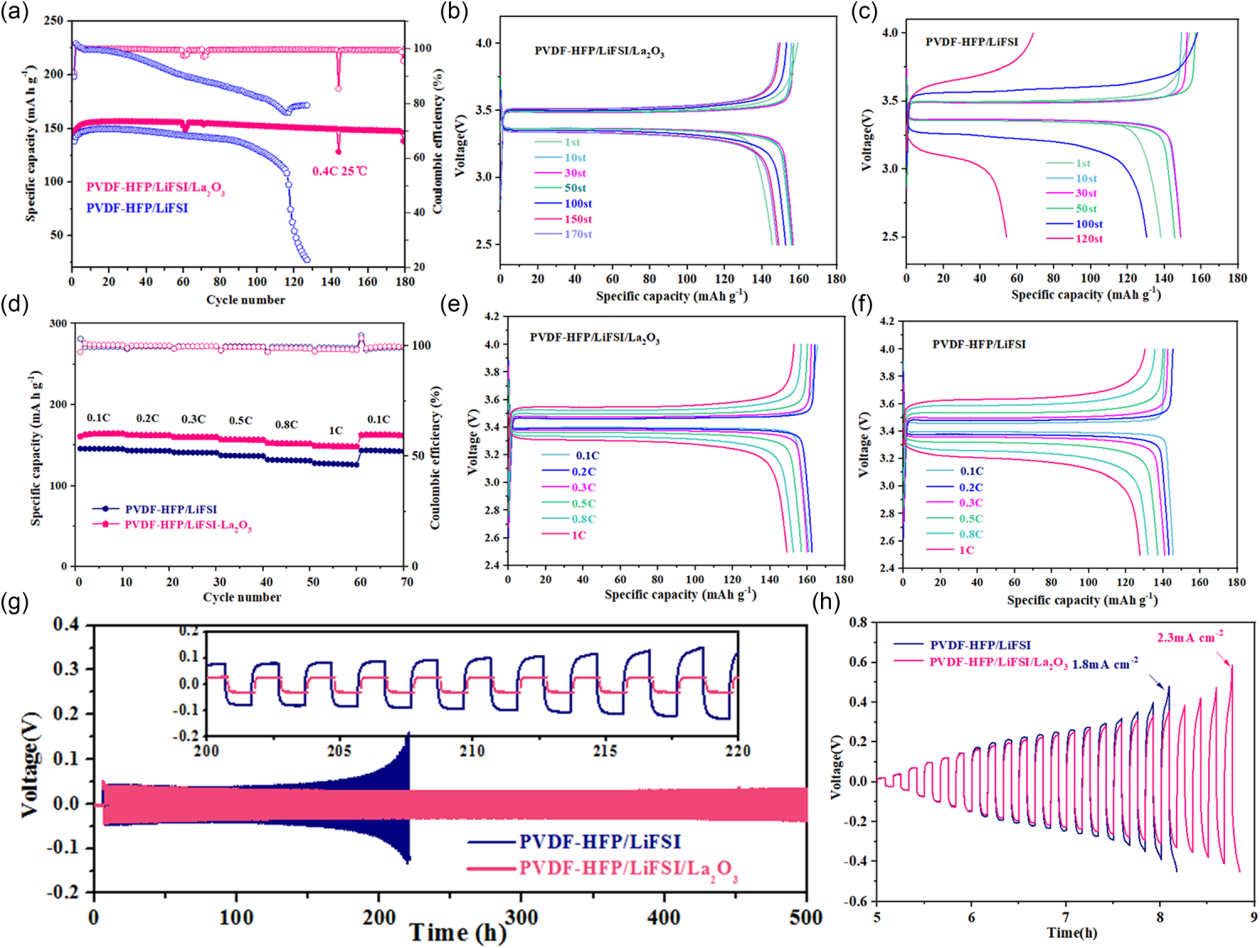
All‐solid‐state LFP||Li coin battery performance. a) Comparison of cycle performance for batteries with PVDF‐HFP/La_2_O_3_ CPE and PVDF‐HFP SE at 0.4C. b,c) Charge/discharge curves at different cycles of LFP||Li batteries using PVDF‐HFP/La_2_O_3_ CPE (b) and PVDF‐HFP SE (c). d) Comparison of rate capacity for batteries with PVDF‐HFP/La_2_O_3_ CPE and PVDF‐HFP SE at 25 °C. e,f) Selective charge–discharge curves at different rates of LFP||Li batteries with PVDF‐HFP/La_2_O_3_ CPE (e) and PVDF‐HFP SE (f). g) Comparison of voltage profiles of Li||Li cells assembled with PVDF‐HFP/La_2_O_3_ CPE and PVDF‐HFP SE at a current density of 0.1 mA cm^−2^ and a cycling capacity of 0.1 mAh cm^−2^. h) CCD of Li||Li cells with PVDF‐HFP/La_2_O_3_ CPE and PVDF‐HFP SE.

To prove the improved electrochemical performance of PVDF‐HFP/La_2_O_3_ CPE in the lithium‐metal batteries, LFP||PVDF‐HFP SE||Li and LFP||PVDF‐HFP/La_2_O_3_ CPE||Li coin cells were assembled. The solid‐state LFP||Li cells fabricated were practically charged and discharged at 0.4C. The corresponding long‐term cycling performance and the charge–discharge curves at different cycles are shown in **Figure** **5**a–c. The LFP||PVDF‐HFP SE||Li cell is damaged after 100 cycles and its discharge capacity decreases from 138 to 97 mAh g^−1^ with average coulombic efficiency of 83%. Benefiting from the addition of La_2_O_3_, the LFP||PVDF‐HFP/La_2_O_3_ CPE||Li cell delivers significantly improved electrochemical stability. The LFP||Li cell exhibits a high reversible capacity of 145 mAh g^−1^ at the first cycle. A high coulomb efficiency of 99.5% and superior cycling stability with 94.5% capacity retention rate can be obtained after 180 cycles. In addition, we also tested the cycling performance of full cells assembled with CPEs containing 30% and 50% La_2_O_3_, respectively (Figure S8 and S9, Supporting Information). The results demonstrate that the cell assembled with the CPE containing 40% La_2_O_3_ has better cycling stability. As presented in Figure 5d–f and S10, Supporting Information, the PVDF‐HFP/La_2_O_3_ CPE‐based battery also shows a much better rate capability. It exhibits a capacity of 164, 162, 160, 156, 151, and 148 mA h g^−1^ at 0.1, 0.2, 0.3, 0.5, 0.8, and 1C, respectively, The voltage profiles of Li||PVDF‐HFP SE||Li and Li||PVDF‐HFP/La_2_O_3_ CPE||Li symmetrical cells are shown in Figure 5g. As shown clearly, the Li||PVDF‐HFP SE||Li symmetric cell displays a short circuit after 200 h, while Li||PVDF‐HFP/La_2_O_3_ CPE||Li symmetric cell exhibits a much longer cycle time and smaller polarization voltage, owing to its better interfacial stability for lithium anode. Critical current density (CCD) is commonly used to evaluate the ability of SEs to suppress lithium dendrites.^[^ ^56^ ^]^ The CCD of the Li||PVDF‐HFP SE||Li cell is increased from 1.1 to 2.2 mA cm^−2^ after the addition of La_2_O_3_ (Figure 5h), indicating that the PVDF‐HFP/La_2_O_3_ CPE could better inhibit the growth of lithium dendrites.

**Figure 6:**
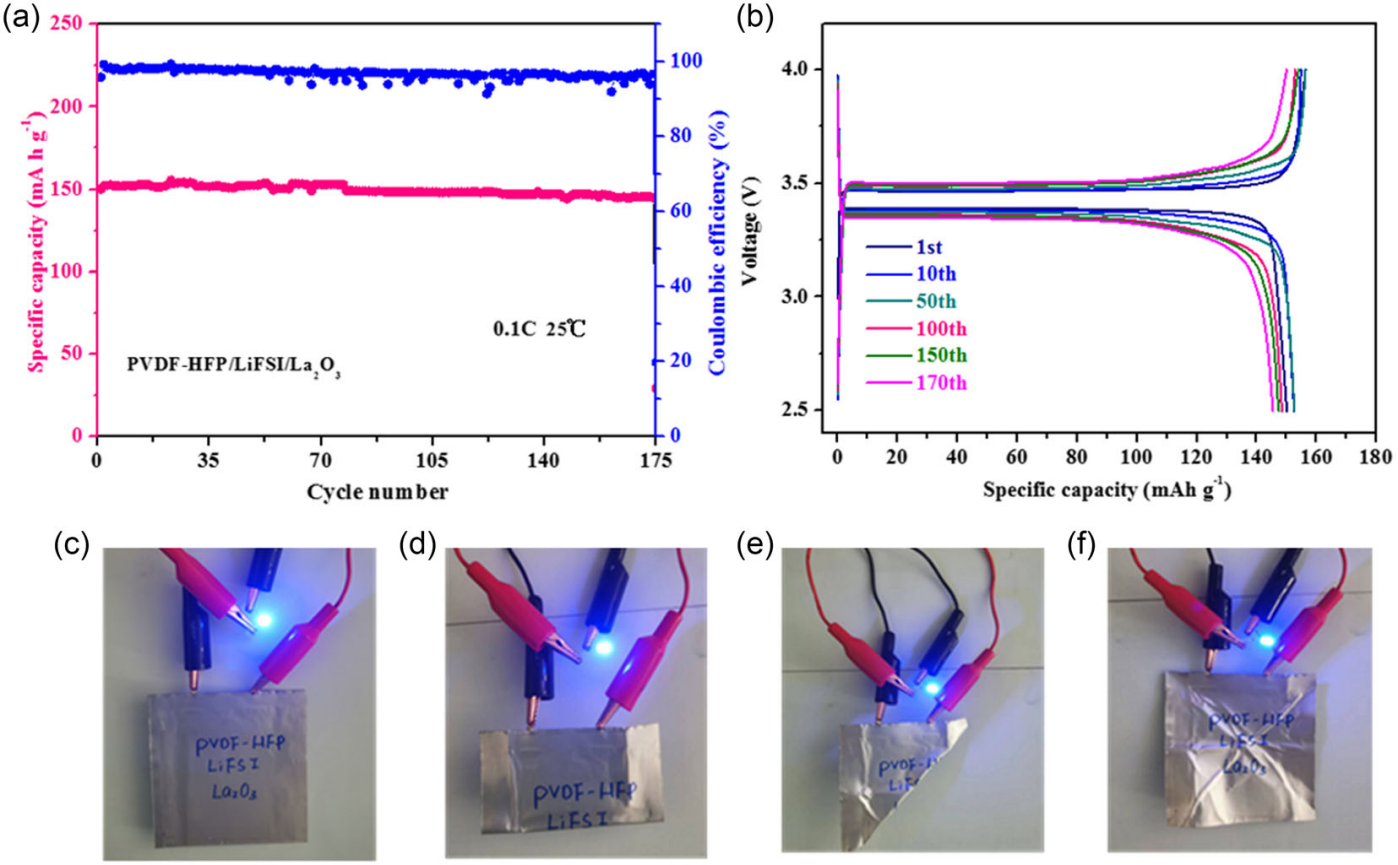
Long‐term cycling performance of the pouch cell using PVDF‐HFP/La_2_O_3_ CPE. a) Cycle performance of LFP||Li operated between 2.5–4.0 V at 0.1C. b) Selective charge–discharge curves after different cycles of LFP||Li. c–f) Images of a pouch cell lighting up a blue LED bulb in different bending conditions.

To further evaluate the flexibility and practical application potential of the PVDF‐HFP/La_2_O_3_ CPE used in lithium‐metal batteries, the LFP||PVDF‐HFP/La_2_O_3_ CPE||Li pouch cells were assembled. The cycle performance of the above cell is evaluated at 0.1C. The battery delivers a high discharge capacity of 150 mAh g^−1^ and a high capacity retention of 96.7% after 170 cycles (**Figure** **6**a,b). The solid‐state pouch cell can successfully brighten the light‐emitting diodes (LEDs) even in the bent cases, as shown in Figure 6c–f, implying the practical application potential of LFP||PVDF‐HFP/La_2_O_3_ CPE||Li SSBs.

## 3 Conclusion

To address interfacial instability caused by the residual DMF solvent in PVDF‐HFP polymer, we developed a new La_2_O_3_ filler into the PVDF‐HFP matrix to form PVDF‐HFP/La_2_O_3_ CPE. Thanks to the reduced crystallinity of PVDF‐HFP, the PVDF‐HFP SE containing 40 wt% La_2_O_3_ nanoparticles exhibited an enhanced ionic conductivity of 1.33 × 10^−3^ S cm^−1^ at 25 °C. In addition, this CPE exhibited higher lithium‐ion transference number and better mechanical strength. More importantly, the La_2_O_3_ nanoparticles had a strong adsorption effect on DMF solvent molecules, which could effectively inhibit their decomposition on the positive or negative interfaces. The assembled cells achieved superior cycling performance and rate capability. All the above results indicate that PVDF‐HFP/La_2_O_3_ CPE has great application potential for advanced lithium‐metal SSBs.

## 4 Experimental Section

4.1

4.1.1

##### Solid Electrolyte Synthesis

La_2_O_3_ was purchased from Shuitian Reagent and was sintered at 900 °C for 12 h before use (Figure S11, Supporting Information). Lithium bis(fluoro sulfonyl) imide (LiFSI) was obtained from Duoduo Chemical Technology Company. We adopted the film‐casting method to prepare composite SE membranes. Typically, PVDF‐HFP (Mw = 400 000, Sigma‐Aldrich) and LiFSI (0.75:1 weight ratio) were first dissolved in *N,N*‐dimethylformamide (DMF, 99.8%, Aladdin) solvents. After magnetically stirring for 12 h, an appropriate quantity of La_2_O_3_ nanoparticles (mass ratio of PVDF‐HFP: La_2_O_3_ = 0.1, 0.2, 0.3, 0.4, 0.5, 0.6) were added to the solution. The mixture was then stirred and ultrasonically processed to obtain a homogeneous electrolyte slurry. Then, the electrolyte slurry was cast onto glass plates by a doctor blade. Finally, the membranes were obtained by further drying at 60 °C for 24 h under vacuum and stored in an argon‐filled glovebox for use. The thickness of the film was 100 μm. The obtained CPEs were denoted as PVDF‐HFP/LiFSI and PVDF‐HFP/LiFSI/La_2_O_3_, respectively.

##### Material Characterization

XRD patterns were collected on an Empyrean‐2 diffractometer to characterize the crystalline structure of La_2_O_3_ filler and obtained CPEs. The morphology of the obtained CPE membranes and cycled lithium metals were observed by a JEOL/JSM‐7610FPlus SEM. FTIR spectral measurements were conducted on a Thermo Scientific Nicolet iS50. TGA using a Netzsch STA 449F3 instrument with a rate of 10 °C min^−1^ from 25 to 600 °C under nitrogen (N_2_) atmosphere to evaluate the thermal stability of CPEs. DSC measurements were performed on a TA Q2000 instrument with a heating rate of 10 °C min^−1^ from 25 to 200 °C under air atmosphere. The stress–strain curve tests were conducted by a universal testing machine (CMT6103). XPS (XPS PHI 5000 VersaProbe) was performed to analyze obtained electrolyte membranes and LFP cathode surface chemistry.

##### Electrochemical Measurements

Electrochemical impedance spectroscopy (EIS) of symmetric SS (stainless steel) ||CPE||SS CR2016 coin cell was measured to evaluate the ionic conductivity *σ* (S cm^−1^) of electrolyte membranes at a Multi Autolab/M204 with a frequency of 1 × 10^−6^ Hz to 0.1 Hz. The ionic conductivity was calculated according to the formula

(1) $$\text{σ = }\frac{L}{R\times S}$$

where *L* (cm) represents the thickness of the CPE Membrane, *R* (Ω) is the bulk resistance, and *S* (cm^2^) is the effective test area. The activation energy *E*_a_ of obtained electrolyte membranes was calculated by the classical Arrhenius equation

(2) $$\text{σ = }A\text{ exp}(‐\frac{E_{a}}{RT})$$

where *A* is the pre‐exponential factor, *R* is the thermodynamic constant, and *T* is the absolute temperature.

The Li^+^ transference number of CPEs was assembled in a Li||CPE||Li coin cell and evaluated by combined DC polarization/AC impedance, and *t*_Li_^+^ was calculated based on the following equation

(3) $$t_{{\text{Li}}^{+}}\text{ = }\frac{I_{s}(\Delta V-I_{0}R_{0})}{I_{0}(\Delta V-I_{s}R_{s})}$$

where *I*_0_ and *I*_s_ are the initial and steady‐state currents values and *R*_0_ and *R*_s_ are the interfacial resistances before and after polarization. Δ*V* is the polarization potential with 10 mV used in this work.

LSV was measured to determine the electrochemical stability window of CPEs by Li||CPE||SS coin cells in the test voltage range of 0 to 6 V with a scan rate of 1 mV s^−1.^ The CV measurements were measured by a Multi Autolab/M204 in LPF||CPEs||Li full cells at a scanning rate of 0.05 mV s^−1^.

##### Battery Testing

The LFP cathode was synthesized by mixing LFP active material, super P, and polyvinylidene fluoride (PVDF, Sigma‐Aldrich) binder with the mass ratio of 8:1:1 in *N*‐methyl‐2‐pyrrolidone (NMP, Aladdin) solvent. Then the slurry was cast on an Al foil current collector and dried at 80 °C for 12 h under vacuum to remove the residual NMP. The average mass loading of LFP active material was about 2.0 mg cm^−2^. Specifically, the mass loading of the cathode active material in the pouch cell was about 1.72 mg cm^−2^, and the mass loading of the cathode active material in the coin cell assembled with PVDF‐HFP/LiFSI SE and PVDF‐HFP/La_2_O_3_ CPE was 2.65 and 2.21 mg cm^−2^, respectively. The charge–discharge tests of cells were conducted on the CT‐4008T Neware battery testing system at 25 °C between 2.5 and 4 V.

##### Density Functional Theory (DFT) Calculation

To probe the interactions between DMF and La_2_O_3_ in CPEs, the adsorption energies were investigated based on DFT calculations performed by the DMol^3^ code. The generalized gradient approximation with the Perdew–Burke–Ernzerhof (PBE) functional and double numerical plus polarization (DNP) basis was used to describe the exchange‐correlation potential. The optimal geometric convergence criteria of energy iteration, force, and atomic displacement were 1.0 × 10^−5^ Ha, 2 × 10^−3^ Ha Å, and 5 × 10^−3^ Å, respectively. The Monkhorst–Pack scheme with 2 × 2 × 1 *k*‐point was used for structural optimization. To reduce the interactions between neighboring layers, the vacuum thickness was set to be more than 15 Å. The formula of adsorption energy is defined as follows.

(4) $$E_{b}=\;E_{\text{total}}-E_{\text{DMF}}-E_{{\text{La}}_{2}O_{3}}$$

where *E*_total_, *E*_DMF_, and *E*_La2O3_ denote the energies of DMF‐La_2_O_3_, DMF, and pristine La_2_O_3_ (0 1 1), respectively.

##### Statistical Analysis

The ionic conductivity data of obtained electrolyte membranes were averaged over at least three replicate measurements. Values were expressed as mean ± SD (*n* = 3), and the values were determined by “Statistics on Rows” using OriginPro 8. The Arrhenius plots were determined via “Fitting Line” using OriginPro 8. The mass loading of LFP active material was the average of three replicates measurements. The better rate capability data were obtained by three independent tests under the same conditions. The values were expressed as mean ± SD (*n* = 4) as presented in Figure S10, Supporting Information, and the values were determined by “Statistics on Rows” using OriginPro 8.

## Conflict of Interest

The authors declare no conflict of interest.

## Supporting information

Supplementary Material

## Data Availability

The data that support the findings of this study are available from the corresponding author upon reasonable request.
