# Supplementary material for: La2O3 Filler's Stabilization of Residual Solvent in Polymer Electrolyte for Advanced Solid‐State Lithium‐Metal Batteries
Source: Small Sci. 2023 Apr 7;3(6):2300017. doi: 10.1002/smsc.202300017 (PMC11935830; doi:10.1002/smsc.202300017)
Supplement: Supplementary file 1 — Supplementary Material [file SMSC-3-2300017-s001.pdf]

Supporting Information

La_2O_3 filler to stabilize residual solvent in polymer electrolyte ensures advanced solid-state lithium-metal batteries

*Yaping Zeng[a], Le Zhao[a], Jiaming Zhang[a], Qiuping Li[a], Dan Sun[a], Yu Ren[b], Yougen Tang[a], Guanhua Jin[c], Haiyan Wang[a,]**

[a]Hunan Provincial Key Laboratory of Chemical Power Sources, College of Chemistry and Chemical Engineering, Central South University, Changsha, 410083, P. R. China.

E-mail: wanghy419@csu.edu.cn; ygtang@csu.edu.cn;

[b]Jiangsu Yeeli Technology Co., Ltd., Wuxi, 214200, P.R. China

[c]College of Energy and Chemical Engineering, Xinjiang Institute of Technology, Aksu, 843100, P.R.China

Supporting Figures

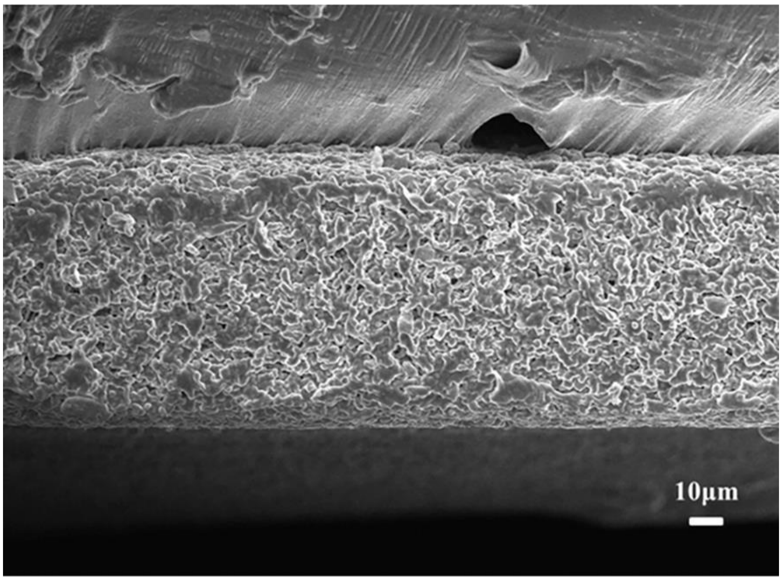

Figure S1. Cross-sectional SEM image of PVDF-HFP/LiFSI/La_2O_3 CPE.

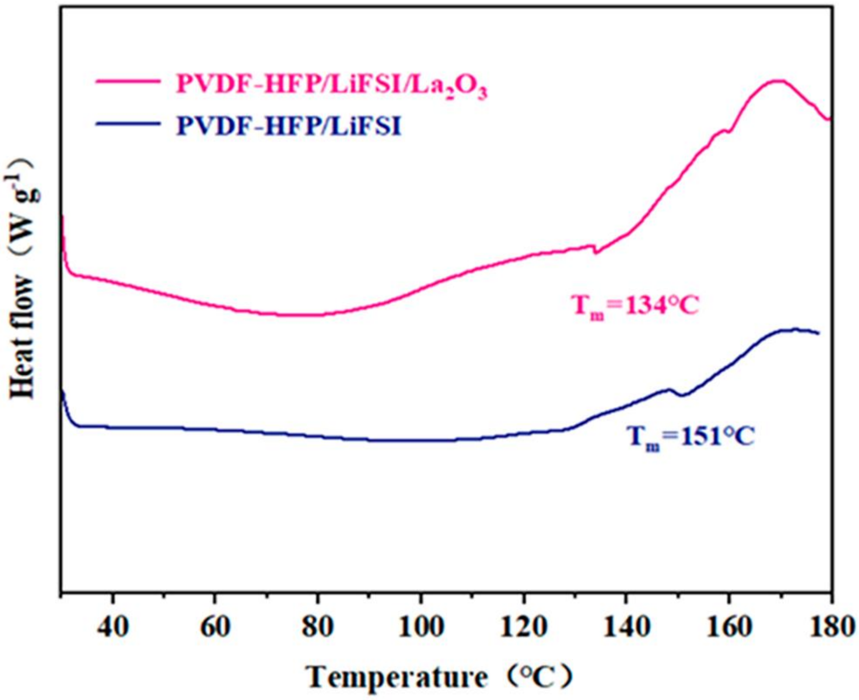

Figure S2. DSC curves of PVDF-HFP/LiFSI SE and PVDF-HFP/LiFSI/La_2O_3 CPE.

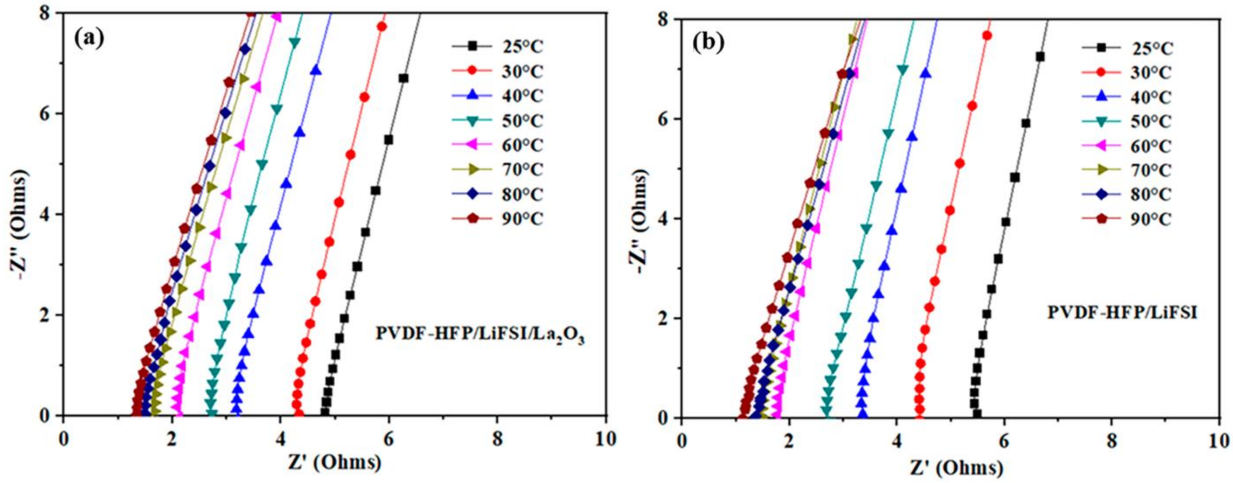

Figure S3. Impendence spectra of a) PVDF-HFP/LiFSI SE and b) PVDF-HFP/LiFSI/La_2O_3 CPE at different temperatures.

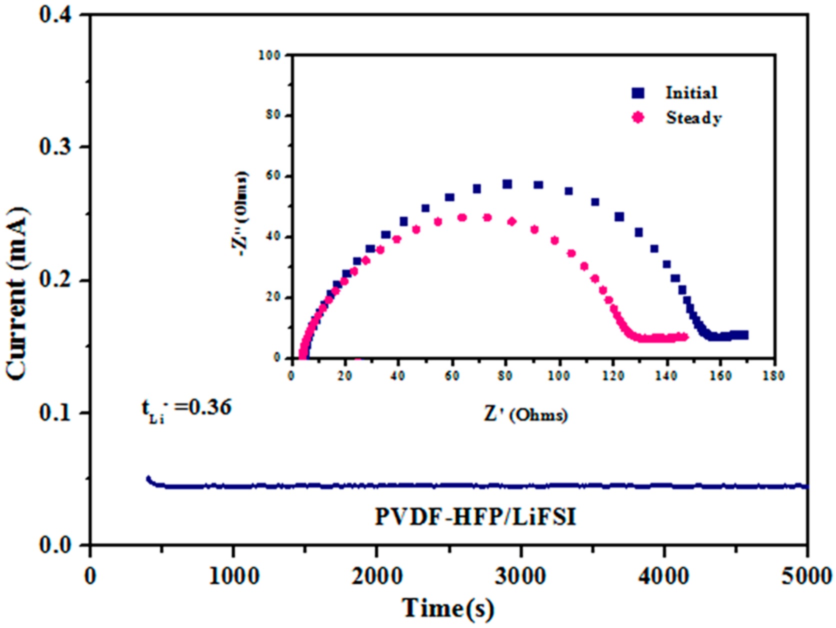

Figure S4. Direct current polarization result for the Li||PVDF-HFP/LiFSI||Li symmetrical cell and its AC impedance spectra before and after polarization (inset).

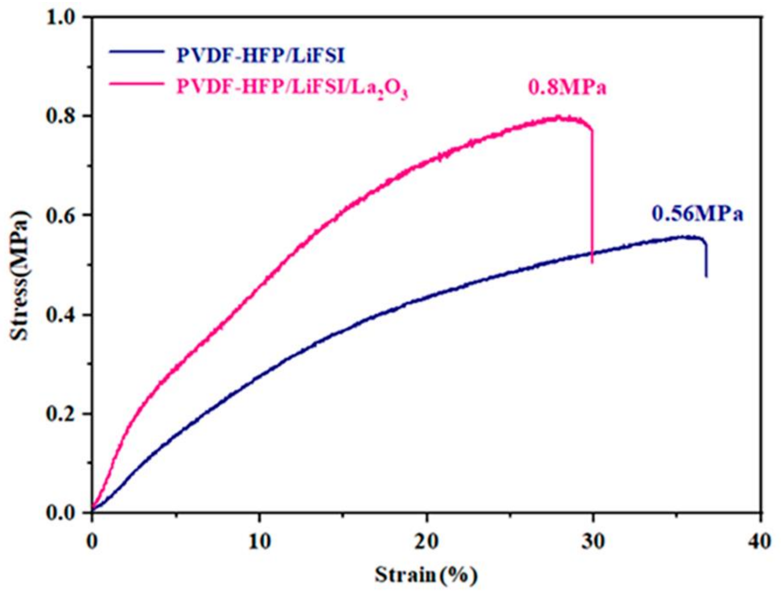

Figure S5. Stress-strain curves of PVDF-HFP/LiFSI SE and PVDF-HFP/LiFSI/La_2O_3 CPE.

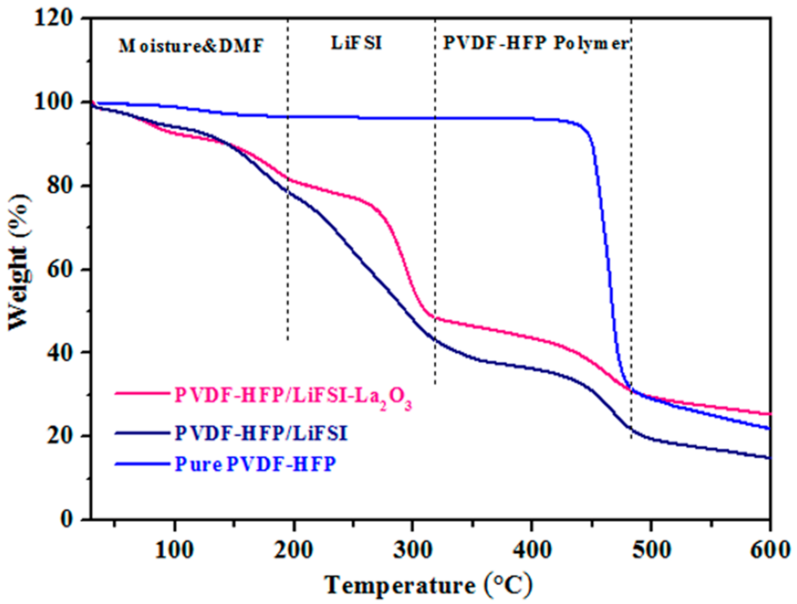

Figure S6. TGA curves of pure PVDF-HFP, PVDF-HFP/LiFSI SE, and PVDF-HFP/LiFSI /La_2O_3 CPE.

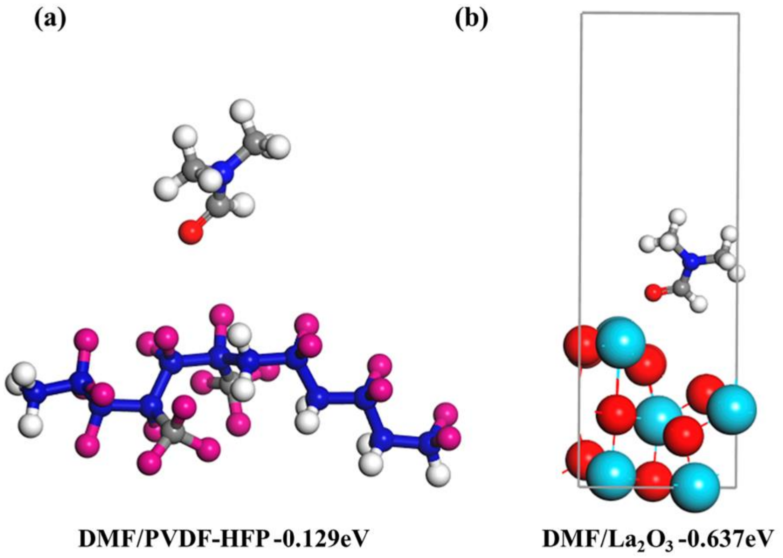

Figure S7. DFT calculation results of adsorption energy. a) PVDF-HFP with DMF. b) La_2O_3 with DMF

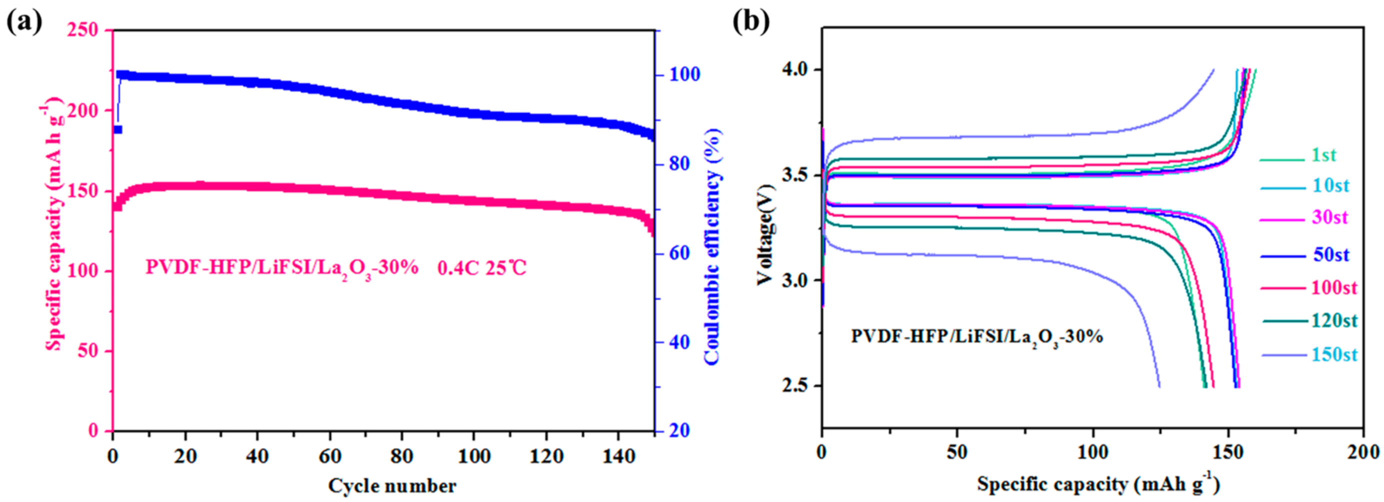

Figure S8. a) The cycle performance for full cell with PVDF-HFP/LiFSI/La_2O_3-30% CPE. b) Charge/discharge curves at different cycles of LFP||Li cell using PVDF-HFP/LiFSI/La_2O_3-30% CPE.

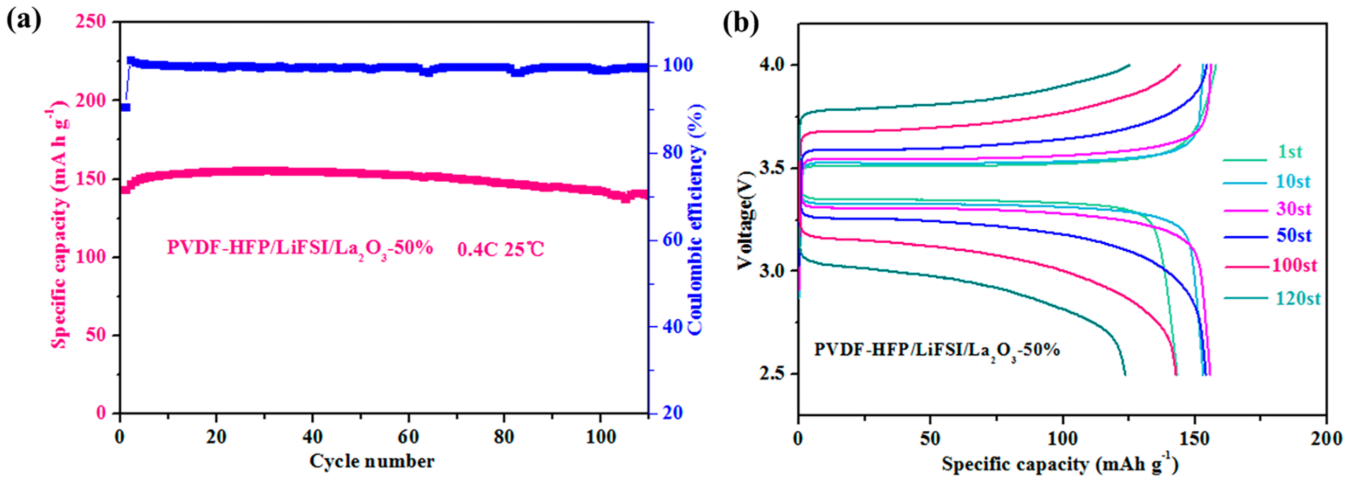

Figure S9. a) The cycle performance for full cell with PVDF-HFP/LiFSI/La_2O_3-50% CPE. b) Charge/discharge curves at different cycles of LFP||Li cell using PVDF-HFP/LiFSI/La_2O_3-50% CPE.

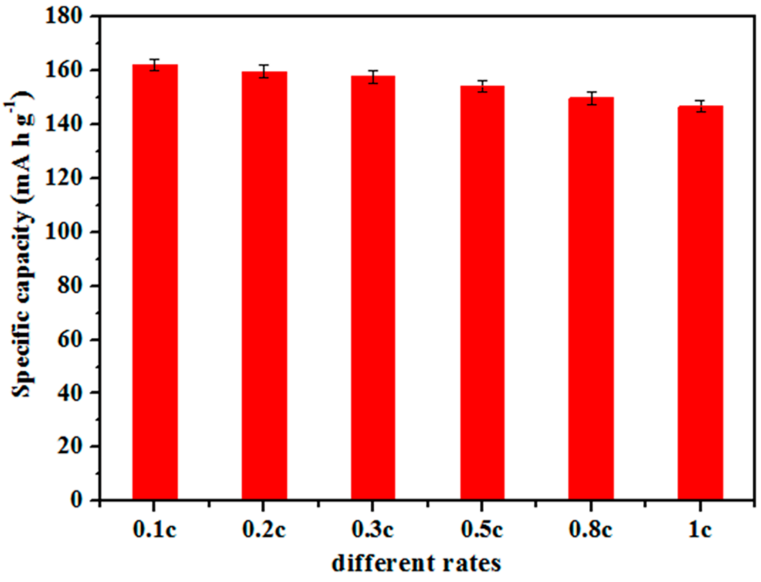

Figure S10. Specific capacities of LFP||PVDF-HFP/La_2O_3 CPE||Li at different rates. Values were expressed as mean ± SD (n=4).

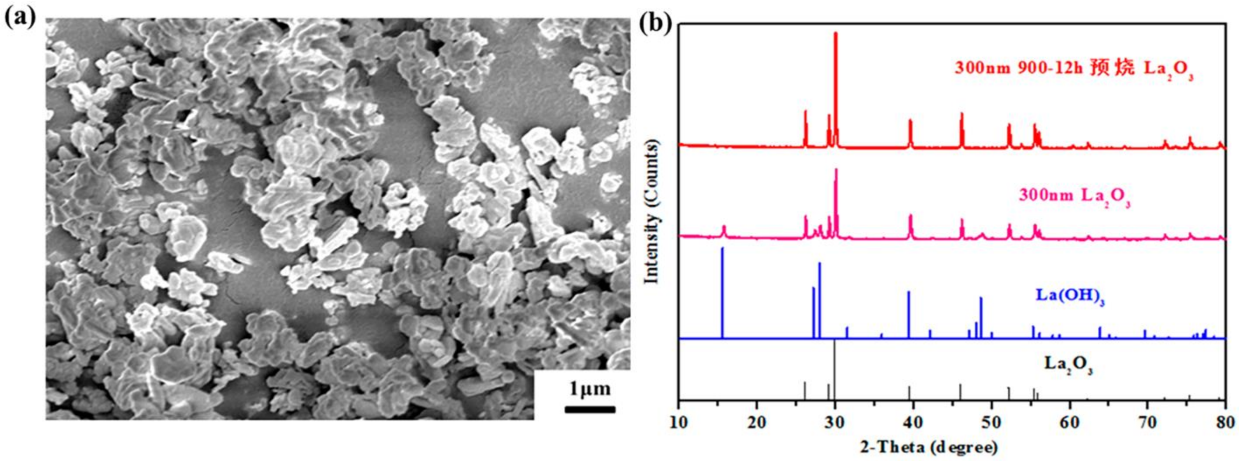

Figure S11. a) SEM image of La_2O_3 nanoparticles. b) XRD patterns of La_2O_3 before and after sintering.

Supporting Tables

Table S1. Comparison of ionic conductivities of different CPEs.

Solid-state electrolyte	Filler	Ionic conductivity (25°C)	Ref.
PVDF-HFP/LiTFSI	$Li_{0.33}La_{0.557}TiO_3$ (LLTO)	1.21×10^{-4} S cm^{-1}	[1]
PVDF-HFP/LiTFSI	$Li_{7-x-3y}Al_yLa_3Zr_{2-x}Ta_xO_{12}$(ATLLZO)	2.686×10^{-4} S cm^{-1}	[2]
PVDF-HFP/LiTFSI	$Li_{1+x}Al_xTi_{2-x}(PO_4)_3$(LATP)	2.3×10^{-4} S cm^{-1}	[3]
PVDF-HFP/LiTFSI	$Li_{6.4}La_3Zr_{1.4}Ta_{0.6}O_{12}$(LLZTO)	1.7×10^{-4} S cm^{-1}	[4]
PVDF-HFP/LiTFSI	$Li_7La_3Zr_2O_{12}$ (LLZO)	9.5×10^{-4} S cm^{-1}	[5]
PVDF-HFP/LiTFSI	$LiAl_2$-LDH	2.2×10^{-4} S cm^{-1}	[6]
PVDF-HFP/LiTFSI	LLZO/CeO_2	4.86×10^{-4} S cm^{-1}	[7]
PVDF-HFP/LiFSI	La_2O_3	1.33×10^{-3} S cm^{-1}	This work

[1] J. Li, L. Zhu, J. Zhang, M. Jing, S. Yao, X. Shen, S. Li, F. Tu, *Int. J. Energy Res.* **2021**, 45, 7663-7674.

[2] J. Zou, X. Gao, X. Zhou, J. Yang, J. Tang, H. Kou, R. Chang, Y. Zhang, *Nanotechnology* **2023**, 34, 155402.

[3] Y. Li, H. Wang, *Ind. Eng. Chem. Res.* **2021**, 60, 1494-1500.

[4] Y. Xu, K. Wang, Y. An, W. Liu, C. Li, S. Zheng, X. Zhang, L. Wang, X. Sun, Y. Ma, *J. Phys. Chem. Lett.* **2021**, 12, 10603-10609.

[5] Y. Li, W. Zhang, Q. Dou, K. W. Wong, K. M. Ng, *J. Mater. Chem. A.* **2019**, 7, 3391-3398.

[6] S. Xia, B. Yang, H. Zhang, J. Yang, W. Liu, S. Zheng, *Adv. Funct. Mater.* **2021**, 31, 2101168.

[7] T. Wang, L. Lu, C. Sun, *Inorg. Chem. Front.* **2022**, 9, 2508-2516.
